# Perception of Iraqi youth towards social and psychological impact of COVID-19

**DOI:** 10.1186/s43045-021-00148-y

**Published:** 2021-10-12

**Authors:** Ameel F. Al Shawi, Riyadh Lafta

**Affiliations:** 1 College of Medicine, University of Fallujah, Fallujah, Iraq; 2 Global Health Department, University of Washington, Seattle, USA

**Keywords:** COVID-19, Perception, Social, Mental, Youth, Iraq

## Abstract

**Background:**

COVID-19 pandemic is recently considered as the most public health challenge with global dramatic changes on different aspects of life and health, including the psychological burden on individuals and communities. A convenience sample of youth (university students) aged 18–24 years was chosen in this cross-sectional study that was conducted during the period from October through December 2020. The questionnaire included questions about COVID-19 and its effect on mental and social wellbeing.

**Results:**

Out of the total 762 young adults who responded, 62% were females, with a mean age of 20.75+2.33; 40.4% of them reported severely impaired social leisure activities; 14.7% expressed severely impaired private leisure activities; and 15.5% had severely impaired ability to form and maintain close relationships. Continuous feeling of nervousness, anxiety, stress, or exaggerated worries about the coronavirus was reported by 18%, while 24.9% felt sad or depressed all the time, and 26.4% of the female respondents had depressive symptoms versus 22.5% males.

**Conclusion:**

The findings of this study indicate that the COVID-19 pandemic had created severe limitations on people’s social activities that may be associated with negative changes in mental condition.

## Background

In December 2019, a group of patients (in Wuhan, Hubei province, China) with pneumonia of unknown cause was confirmed to be infected with a novel coronavirus, known as 2019-nCoV that had not been previously detected in humans or animals. In March 2020,pandemic” [1–3]. This pandemic has been labeled as the most public health challenge in the last four decades because, in addition to the high rates of morbidity and mortality, it was associated with global dramatic changes in life including lifestyle, traveling, communications, and financial issues. All these changes created noticeable effects on different aspects of life and health, causing more psychological burdens on individuals and communities [4, 5].

In Iraq, which is known to have a vulnerable health system that was further weakened by years of war and sectarian conflicts, the first case reported was an international student in Najaf governorate on February 24, 2020, followed 3 days later by a second (national) case, which was followed by a series of cases in different provinces of the country. On March 17, 2020, the authorities imposed the lockdown, suspended schools, and closed mosques, malls, shops, casinos, and other crowded places. The fear of getting COVID-19 infection resulted in a strict limitation of movement and communication impairment in work that appeared to be associated with serious psychological distress such as anxiety and depression [6, 7]. Exposure (or fear of exposure) to the virus is an important risk factor of psychiatric symptoms as threat perception could be intensified for some people and reflect their fear of being exposed (or their relatives) to the virus [8, 9].

This study aims to help understand the social, behavioral, and psychological impacts of COVID-19 on Iraqi youth.

## Methods

### Study setting and study population

This descriptive cross-sectional study (with an analytic element) was conducted during the period from October through December 2020, on a convenience sample of young adults aged 18–24 years that was collected from different Iraqi governorates. Data were collected online (a web-based survey), the questionnaire form was distributed electronically to the target population in different Iraqi governorates.

### Assessment tools

The questionnaire consisted of a socio-demographic section that included age, gender, level of education, marital status, and residence. The second section (screening tool) included questions about COVID-19 and its possible effect on social life and behavior in order to assess the level of impairment the pandemic has caused. The questionnaire was borrowed from the “Australian National COVID-19 mental health, behavior and risk communication survey” [10] with some modifications to suit the Iraqi culture. These questions included ability to work, ability to study, home management (cleaning, tidying, shopping, cooking, looking after home or children, paying bills), and also social leisure activities (with other people), such as parties, bars, clubs, outings, visits, and home entertainment, in addition to private leisure activities (activities that are done alone) such as reading, gardening, sewing, and walking alone, on a scale ranging from “not at all impaired” to “very severely impaired” during the last 6 months.

The last group of questions was to illustrate the effect of COVID-19 on participants’ mental health such as feeling nervous, anxious, stressed, or worried; having trouble sleeping or nightmares related to the coronavirus; and feeling sad or depressed during the last 6 months, with 5° Likert scale (from not at all to all of the time) [11]. The questionnaire was translated from English to Arabic, then to English to enhance validity. Data were collected electronically using the survey monkey program.

### Ethical consideration

At the top of the questionnaire form, a paragraph was added that refers to the consent of the participants to be involved in the survey after explaining to them its purpose and assuring the confidentiality of the information they give. The final ethical approval for the implementation of the study was obtained from the Scientific and Ethical Committee of the Medical College/University of Fallujah.

### Statistical analysis

Data were presented as numbers and percentages. Chi-square test was used to assess the significance of association between variables with a cutoff point of *p* value < 0.05 to be considered as significant.

## Results

A total of 762 youth was collected from different governorates, mainly Baghdad (50%), 66.1% of whom were females and 93.1% were single, and their mean age was 20.75 + 2.33—more details about the socio-demographic characteristics are shown in Table 1 which also demonstrates that 4% of the respondents were diagnosed as having coronavirus infection, 22.2% had a family member or know someone who had coronavirus, and 15.6% reported having a family member or knowing someone who died from coronavirus.

**Table 1:** Socio-demographical characteristics of the sample

		No.	%
Gender	Female	473	62.1
	Male	289	37.9
		762	100
Marital status	Single	710	93.1
	Married	52	6.8
Age (mean+SD)	20.75+2.33		
I was diagnosed positive with coronavirus		31	4
I had symptoms but was never tested		24	3.1
Isolated or quarantined due to possible exposure		15	19.6
I have a family member or know someone who was diagnosed positive		169	22.2
I have a family member or know someone who died from coronavirus		119	15.6

Table 2 shows that 40.4% of the respondents reported severely impaired social leisure activities (group activities), such as parties, celebrations, outings, visits, or home entertainment; 14.7% expressed severely impaired private leisure activities; and 15.5% of the participants had severely impaired ability to form and maintain close relationships during the period of the pandemic.

**Table 2:** Levels for social impairment caused by coronavirus pandemic

	0	1	2	3	4
	(Not at all)	(little)	(Somewhat)	(Frequently)	Severely impaired
	No. (%)	No. (%)	No. (%)	No. (%)	No. (%)
Ability to work	247 (32.4)	117 (15.5)	176 (23.1)	108 (14.12)	114 (15)
Ability to study	193 (25.3)	122 (16)	156 (20.5)	137 (18)	154 (20.2)
Home management	281 (36.9)	139 (18.2)	158 (20.7)	105 (13.8)	79 (10.4)
Social leisure activities	169 (22.2)	79 (5.8)	88 (11.5)	118 (15.5)	308 (40.4)
Private leisure activities	311 (40.8)	145 (19)	124 (16.3)	70 (9.2)	112 (14.7)
Ability to form and maintain close relationships	291 (38.2)	137 (18)	143 (18.8)	37 (9.6)	118 (15.5)

Table 3 clarifies that 18% of the respondents reported a continuous feeling of nervousness, anxiety, stress, or exaggerated worries about coronavirus, while 24.9% are feeling sad or depressed all the time; 35.8% of the participants had anxiety about COVID-19 in (most/all of the time); and 41.2% reported COVID-related depression in (most/all of the time).

**Table 3:** Statistics of mental health symptoms associated with COVID-19 pandemic

	Not at all impaired	Little of time	Some time	Most of the time	All the time	P*
	*n* (%)	*n* (%)	*n* (%)	*n* (%)	*n* (%)	
Feel nervous, anxious, stressed	141 (18.5)	135 (17.7)	213 (28)	136 (17.8)	137 (18)	
Female	86 (18.2)	82 (17.3)	127 (26.8)	83 (17.5)	95 (20.1)	0.42
Male	55 (19)	53 (18.3)	86 (29.8)	53 (18.3)	42 (14.2)	
Have trouble sleeping or nightmares	558 (73.2)	97 (12.7)	52 (6.8)	25 (3.3)	30 (3.9)	
Female	347 (73.4)	58 (12.3)	33 (7)	17 (3.6)	18 (3.8)	0.95
Male	211 (73)	39 (13.5)	19 (6.6)	8 (2.8)	12 (4.2)	
Feel sad or depressed	164 (21.5)	146 (19.2)	138 (18.1)	124 (16.3)	190 (24.9)	
Female	102 (21.5)	88 (18.6)	77 (16.3)	81 (17.1)	125 (26.4)	0.38
Male	62 (21.5)	58 (20.1)	61 (21.1)	43 (14.9)	65 (22.5)	

*Chi-square was used

The results also revealed that 26.4% of the female respondents had depression symptoms (all of the time) associated with COVID-19 versus 22.5% males with no significant gender difference (Table 3).

## Discussion

Assessing the hazards of an infectious disease is usually a vital concern in epidemiology [12]. However, the rapid spread of an infectious disease is usually associated with anxiety, fear, psychological distress, and other mental symptoms [13].

The findings revealed a negative impact of COVID-19 on the social and mental wellbeing of youth. More than half of the respondents reported some impairment of social activities, and many of them reported “severely impaired” leisure activities; this could be attributed in part to the panic of being in contact with the “probably” infected persons in overcrowded places, and, on the other hand, to the lockdown that was imposed by the health and political authorities for more than 3 months. These findings are consistent with what was reported in some other studies [5, 14, 15].

The incidence of mental symptoms is known to be lower in youth compared to other age categories. Meta-analysis of the prevalence of anxiety and depression among adolescents and young adults generated a pooled prevalence estimate of 19.1% and 14.3%, respectively [16]; however, the current study showed a higher incidence of anxiety and depressive symptoms among youth—this could be attributed to the psychological consequences of the pandemic which created a feeling of panic from the sequels of COVID-19 especially after the accelerating registration of deaths.

Men are usually less vulnerable to suffer from mental symptoms than women [17, 18]; however, the interesting finding in the current study was that the prevalence of mental symptoms in males is approximating that in females with no significant difference, this could be attributed to the effect of the prolonged lockdown and strict limitation of movement that was not experienced by males (especially youth—the most active age group) before the time of pandemic [19] although anxiety and nervousness were a bit more in women compared to men. The prolonged exposure of the Iraqis to continuous wars, sanctions, armed conflicts, and wide spectrum of violence for more than four decades [20, 21] might have a deep influence on their thoughts, feelings, and behaviors.

The feeling of anxiety (most/all of the time) was higher than what was reported among samples of university students in the United Arab Emirates, ^5^ Australia, ^6^ and in South-west Ethiopia [22]. This could be explained by differences in culture, instruments used, and sampling techniques.

The importance of this study comes from the validity of its genuine subject; however, the findings may be difficult to interpret because of the potential limitation of the sample size, due to the relatively low response rate attributed to the sensitivity of the subject and people avoidance to share information related to their psychological condition. Despite this limitation, these data allow important conclusions to be drawn about the social and mental burden of this newly emerging disease more broadly.

## Conclusions

The findings of this study indicate that, so far, the COVID-19 pandemic had created severe limitations on social activities of the population that might be associated with negative changes in mental condition and could lead to a sort of social and psychological withdrawal, the duration and remote consequences of which are difficult to predict. Further national surveys with larger samples and in-depth analyses are required to more elaborate the effect of COVID-19 on the social and mental health of the Iraqi population.

## Data Availability

The datasets that were generated during and/or analyzed during the current study are available from the corresponding author on request.

## Abbreviation

COVID-19: Coronavirus disease 2019
